# Supplementary figures and images for: Deregulation of LRSAM1 expression impairs the levels of TSG101, UBE2N, VPS28, MDM2 and EGFR
Source: PLoS One. 2019 Feb 6;14(2):e0211814. doi: 10.1371/journal.pone.0211814 (PMC6364939; doi:10.1371/journal.pone.0211814)

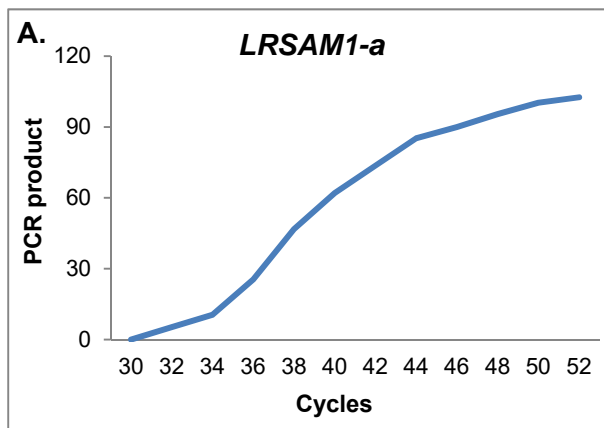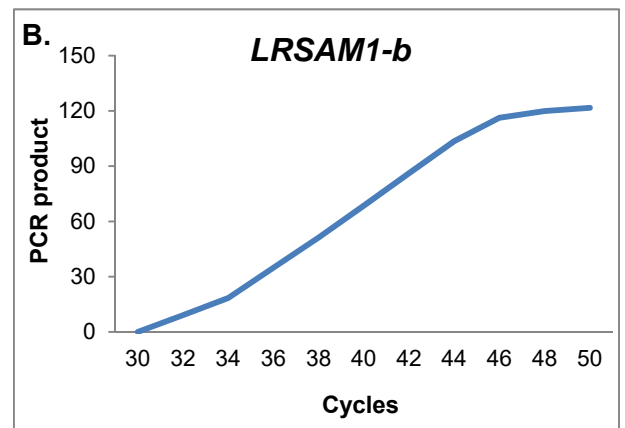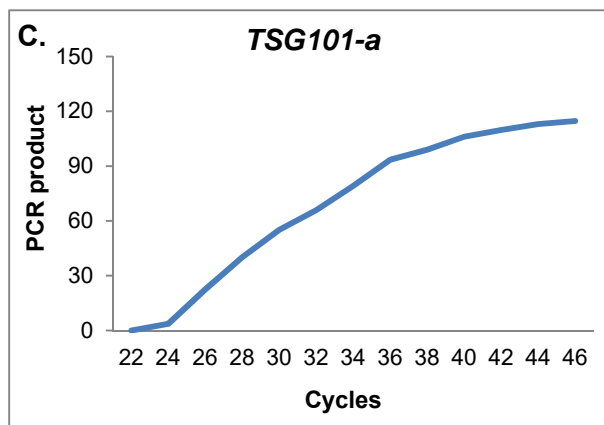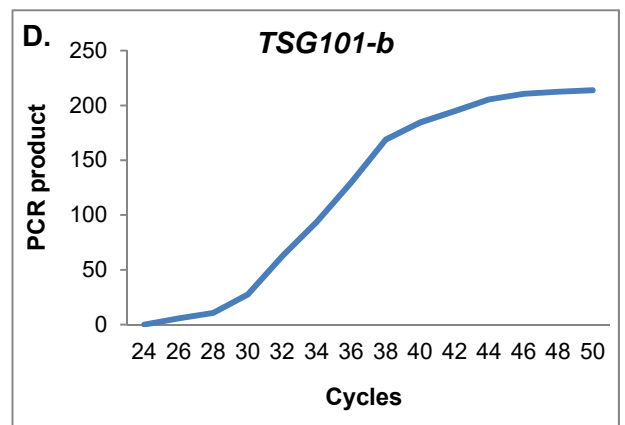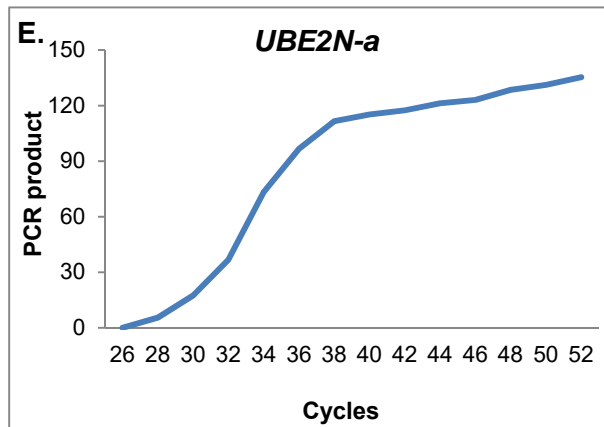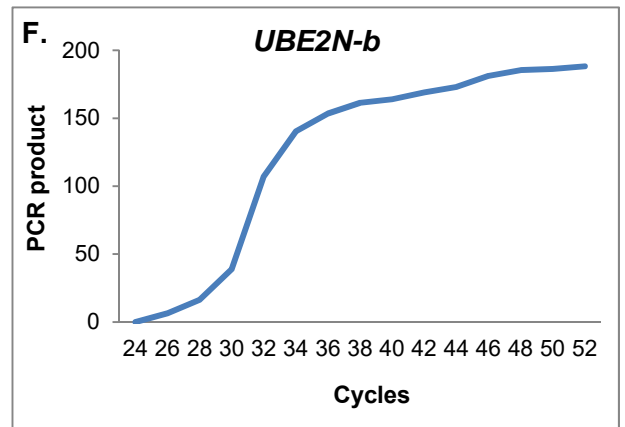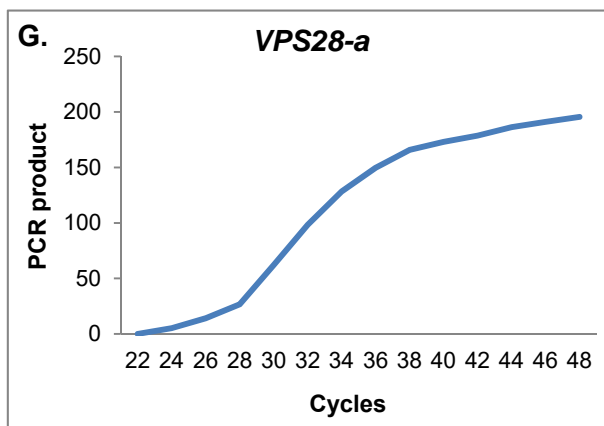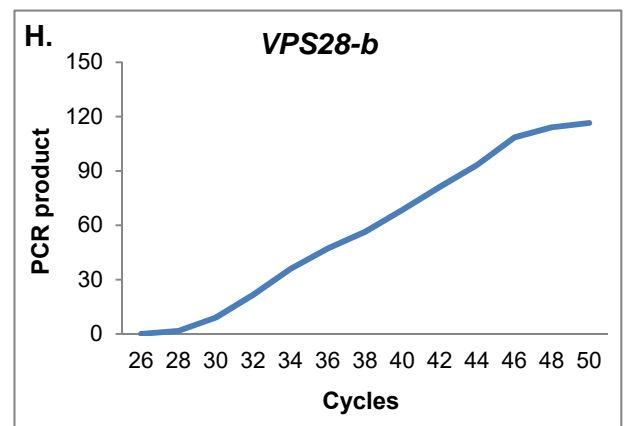

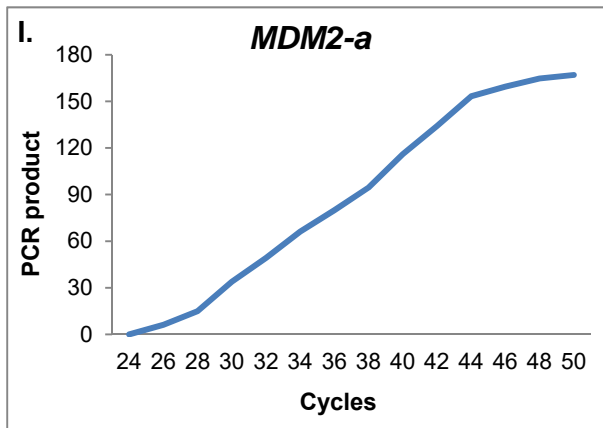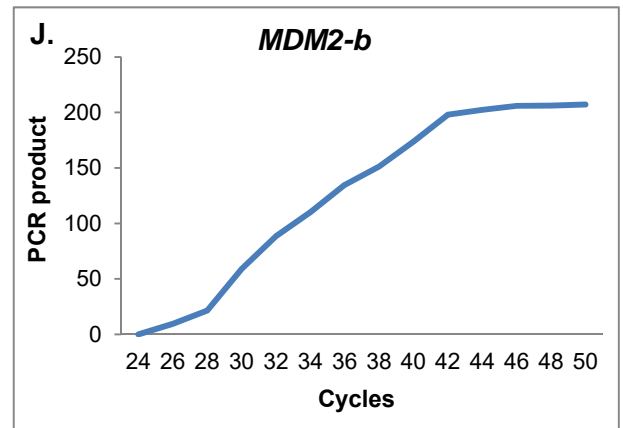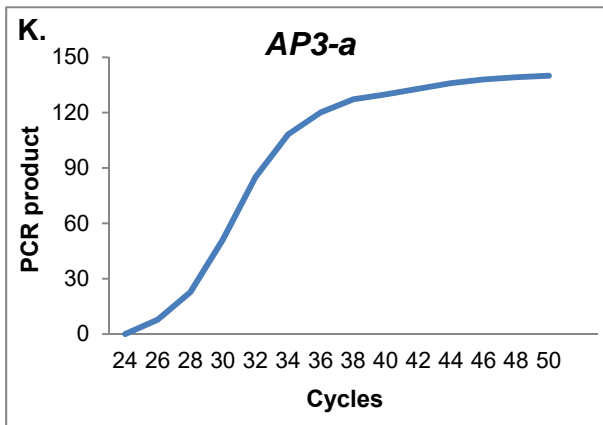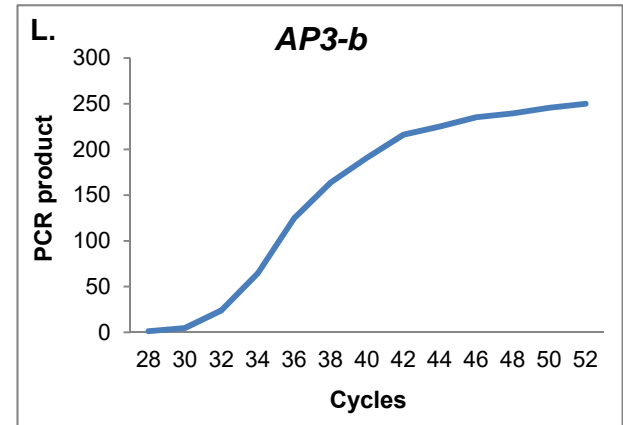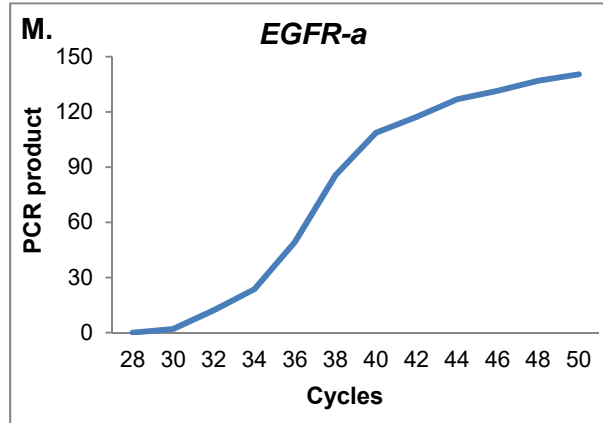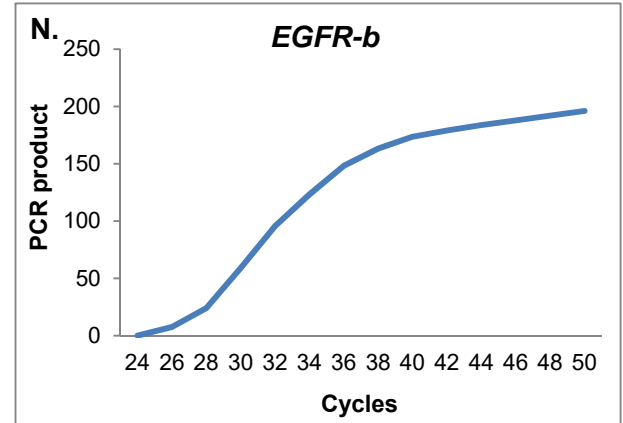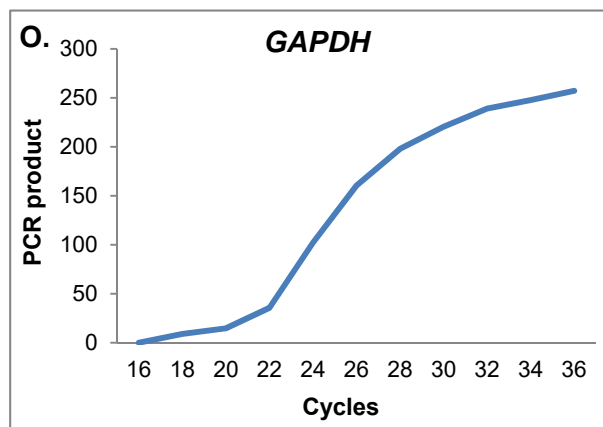

Supplement: S1 Fig — Standard curves were used to determine the cycles of the exponential phase for each set of primers used in the RNA expression analysis. (A) LRSAM1-a: 37 cycles, (B) LRSAM1-b: 38 cycles, (C) TSG101-a: 33 cycles, (D) TSG101-b: 33 cycles, (E) UBE2N-a: 31 cycles, (F) UBE2N-a: 34 cycles, (G) VPS28-a: 32 cycles, (H) VPS28-b: 35 cycles, (I) EGFR-a: 37 cycles, (J) EGFR-b: 33 cycles, (K) MDM2-a: 33 cycles, (L) MDM2-b: 33 cycles, (M) AP3-a: 31 cycles, (N) AP3-b: 36 cycles (O) GAPDH: 24 cycles. (PDF) [file pone.0211814.s001.pdf]
